# miR-223: A Key Regulator in the Innate Immune Response in Asthma and COPD

**DOI:** 10.3389/fmed.2020.00196

**Published:** 2020-05-19

**Authors:** Mirjam P. Roffel, Ken R. Bracke, Irene H. Heijink, Tania Maes

**Affiliations:** ^1^Laboratory for Translational Research in Obstructive Pulmonary Diseases, Department of Respiratory Medicine, Ghent University Hospital, Ghent University, Ghent, Belgium; ^2^Departments of Pathology and Medical Biology and Pulmonology, Groningen Research Institute for Asthma and COPD, University of Groningen, University Medical Center Groningen, Groningen, Netherlands

**Keywords:** asthma, COPD, miRNAs, miR-223, inflammation

## Abstract

Asthma and Chronic Obstructive Pulmonary Disease (COPD) are chronic obstructive respiratory diseases characterized by airway obstruction, inflammation, and remodeling. Recent findings indicate the importance of microRNAs (miRNAs) in the regulation of pathological processes involved in both diseases. MiRNAs have been implicated in a wide array of biological processes, such as inflammation, cell proliferation, differentiation, and death. MiR-223 is one of the miRNAs that is thought to play a role in obstructive lung disease as altered expression levels have been observed in both asthma and COPD. MiR-223 is a hematopoietic cell–derived miRNA that plays a role in regulation of monocyte-macrophage differentiation, neutrophil recruitment, and pro-inflammatory responses and that can be transferred to non-myeloid cells via extracellular vesicles or lipoproteins. In this translational review, we highlight the role of miR-223 in obstructive respiratory diseases, focusing on expression data in clinical samples of asthma and COPD, *in vivo* experiments in mouse models and *in vitro* functional studies. Furthermore, we provide an overview of the mechanisms by which miR-223 regulates gene expression. We specifically focus on immune cell development and activation and involvement in immune responses, which are important in asthma and COPD. Collectively, this review demonstrates the importance of miR-223 in obstructive respiratory diseases and explores its therapeutic potential in the pathogenesis of asthma and COPD.

## Introduction

Worldwide, the prevalence of people with chronic respiratory disease is increasing. The most common chronic inflammatory airway diseases are asthma and chronic obstructive pulmonary disease (COPD), with a prevalence of 358 million asthma patients and 174 million COPD patients, respectively (1). Over the last years, it has become clear that both diseases are complex and heterogeneous with different underlying processes (2, 3).

Asthma affects the large and small airways leading to symptoms such as shortness of breath, wheezing, coughing, and chest tightness. The cause of asthma is unknown, however there are several risk factors influencing the development and severity of asthma. The most common risk factors are host factors (including genetics) and environmental factors (allergens, viral/microbial infections, air pollutants, smoke) (4).

Asthma can be divided in several phenotypes depending on presence of allergy, inflammatory profiles, and age of onset (2). Allergic asthma is the most common form of asthma, where allergens such as house dust mite, pollen, and pet dander trigger a type 2-driven inflammatory response in the lungs. This leads to airway remodeling, with increased smooth muscle mass, airway hyperresponsiveness, and mucus hypersecretion (4). Allergic asthma, which generally develops early in life, is characterized by the presence of allergen specific immunoglobulin E, elevated airway infiltration of eosinophils and T helper 2 (Th2) lymphocytes and increased levels of type 2 cytokines e.g., IL-5 and IL-13 (2). Type 2 inflammation can also occur in non-allergic eosinophilic asthma, which develops later in life, is often more severe and is associated with increased numbers of type 2 innate lymphoid cells (ILC2s) that produce IL-5 and IL-13 in response to epithelial damage (5). Other, non-type 2 asthma phenotypes are characterized by elevated sputum neutrophils numbers (called neutrophilic asthma) or by airway remodeling that is not accompanied by higher sputum levels of eosinophils and neutrophils (called paucigranulocytic asthma) (2). The clinical manifestations of asthma can be controlled by inhaled corticosteroids (ICS), combined with bronchodilators in the majority of asthma patients, while a subset of patients with severe asthma is insensitive to ICS (6, 7).

COPD affects around 10% of the world's population and is the third leading cause of death worldwide (8). In COPD, chronic inflammation mainly affects the small airways leading to symptoms such as shortness of breath, chronic cough and/or wheezing (6). It is a heterogeneous disease characterized by irreversible airflow limitation and accelerated lung function decline caused by pulmonary inflammation, chronic bronchitis, (small) airway remodeling and/or destruction of alveolar tissue (emphysema) (9). The major risk factor for developing COPD is chronic exposure to noxious particles and gases such as cigarette smoke, air pollution and occupational exposures. In COPD, the exposure to those noxious particles and gases leads to pulmonary infiltration of predominantly neutrophils and CD8^+^ T lymphocytes. Furthermore, macrophages play a major role in the inflammatory response in COPD, upon activation macrophages release several cytokines and matrix metalloproteases (10). In the lung of severe COPD patients increased B cell counts and increased number and size of B-cell rich lymphoid follicles have been found (11). The major treatment in COPD patients is long-acting bronchodilators and ICS. However, COPD patients are less sensitive to ICS compared to allergic asthma patients (10). In asthma and COPD, current treatments relieve symptoms and prevent exacerbations, however, they cannot counteract the underlying disease process.

MicroRNAs (miRNAs) are small, non-coding RNA molecules with a length of 21–25 nucleotides. MiRNAs can control gene expression by targeting specific mRNAs for degradation or translational repression. MiRNAs bind to a specific sequence at the 3′UTR of the target messenger RNA (mRNA). The complementary sequences between miRNA and mRNA are usually not fully overlapping, implicating that each miRNA can regulate hundreds of target genes and that several miRNAs can target the same mRNAs (12).

MiRNAs are involved in multiple biological processes including inflammation, cell proliferation, differentiation, and death and may thus regulate various pathological processes (13). Indeed, miRNAs have been implicated in many diseases. Furthermore, miRNAs are present in bodily fluids due to active secretion from living cells in microvesicles, secretion from cells with RNA-binding proteins or leakage of microvesicles from death cells (14). Due to stability and high expression of miRNAs in bodily fluids, they can be potential biomarkers in asthma and COPD. As mentioned above, the development of asthma and COPD is the consequence of an interaction between genetic and environmental factors. By regulating gene expression, miRNAs may constitute an important link between the factors involved in the development of obstructive lung disease.

In this review, we will focus on the potential role of miR-223 in the pathogenesis of asthma and COPD, since miR-223 is differentially expressed in various tissues from asthma patients compared to healthy controls and is among the highest differentially expressed miRNA in COPD patients. Furthermore, miR-223 has a crucial role in innate immunity, myeloid cell differentiation, and cell homeostasis and has several targets that are involved in pathways implicated in the pathogenesis of both asthma and COPD. Finally, miR-223 is easily detectable in multiple human samples (e.g., sputum, bronchial biopsies, and blood) and could therefore serve as a potential biomarker.

## Origin of miR-223 and Role in Haematopoiesis

Although the role of miR-223 in hematopoietic cell development and innate and adaptive immune responses has recently been extensively reviewed by others (15), we shortly summarize the most important findings with regard to the role of miR-223 in hematopoiesis. MiR-223 is transcribed from an independent promoter located on the X chromosome and is mainly expressed by hematopoietic cells (16). Whereas, levels of miR-223 are low in pluripotent hematopoietic stem cells and common myeloid progenitors, miR-223 expression is induced upon myeloid differentiation (17). In humans, the expression of miR-223 is controlled by two transcriptional factors that compete for binding to the miR-223 promoter, namely nuclear factor I A-type (NFI-A) and CCAAT enhancer protein α (C/EBPα). Under resting conditions, NFI-A is bound to the miR-223 promoter, maintaining low expression of miR-223. During granulocytic differentiation, NFI-A is released from the miR-223 promoter and replaced by C/EBPα, resulting in upregulation of miR-223 expression (16). Interestingly, one of the target genes of miR-223 is NFI-A, implicating that upregulation of miR-223 dampens the expression of NFI-A, resulting in a positive feedback loop. In line with a role of miR-223 in myeloid differentiation, overexpression of miR-223 induces the monocytic and granulocytic differentiation marker CD11b, while inhibition of miR-223 was shown to reduce the expression of CD11b in promyelocytic leukemia cells (16). In addition to the importance in myeloid differentiation, miR-223 is involved in erythropoiesis by dampening the gene expression and protein translation of LIM-only protein 2, a positive regulator of erythropoiesis (18, 19).

MiRNA profiling in human blood demonstrated that miR-223 is expressed in hematopoietic stem cells, granulocytes, dendritic cells and monocytes, while lower levels of miR-223 were also found in naïve and memory T cells (16, 20, 21). In induced sputum high expression of miR-223 was measured in monocytes, macrophages and neutrophils (22). Furthermore, *in situ* hybridisation in human bronchial biopsies showed that miR-223 expression was mainly expressed in neutrophils localized in the lamina propria (22).

In this review, we will use findings of *in vivo* murine models to provide mechanistic insight into how miR-223 can contribute to the pathogenesis of asthma and COPD. In mice, the regulation of miR-223 expression is slightly different compared to humans. The myeloid differentiation factors PU.1 together with C/EBPβ enhance the activity of the miR-223 promoter, whereas erythroid transcription factor GATA binding protein 1 represses the miR-223 promoter (23). Depletion of miR-223 in a mouse model does not result in apparent developmental abnormalities, however granulocyte hyperplasia has been observed in the bone marrow (17). The increased numbers of neutrophils in miR-223 deficient mice display morphological changes, including nuclear hypersegmentation and blebbing and are hyperactivated as demonstrated by their increased superoxide production (17). Similar to humans, granulocyte-monocyte progenitors express low levels of miR-223 in blood and bone marrow obtained from mice, but during myeloid differentiation the expression levels of miR-223 are increased. Expression of miR-223 is highest in mature neutrophils, whereas B- and T-cells barely express miR-223 (17, 24, 25).

In summary, the expression of miR-223 is controlled by both enhancers and repressors, is low in haematopoietic progenitor cells and increased during granulopoiesis, indicating its importance in the control of cell homeostasis.

## Expression of miR-223 in Asthma and COPD

As described above, miR-223 is mainly expressed in myeloid cells and may play a role in innate immunity. Therefore, several studies investigated the expression of miR-223 in asthma and COPD patients (Table 1). The first report on miRNA profiling in asthma patients, where miR-223 was mentioned, was performed in bronchial biopsies of eight mild atopic asthmatic patients and eight healthy controls using a miRNA array. No difference in miRNA expression was observed between asthma patients and healthy controls (26). However, miR-223 was one of highest expressed miRNAs in biopsies and was shown to be expressed in macrophages (26). No differences in miR-223 expression were found in an additional study on bronchial biopsies from 12 mild and 12 severe asthma patients compared to healthy controls using quantitative real time polymerase chain reaction (27), which may be due to the low sample size. In larger cohorts and more specific compartments differences in miR-223 expression between asthma patients and healthy controls were identified. MiRNA profiling in bronchial brushings obtained from 16 steroid naive asthmatics compared to 12 healthy controls demonstrated higher expression of miR-223 in asthma patients compared to healthy controls (28). Two independent asthma cohorts showed that miR-223 is higher expressed in induced sputum supernatant of severe asthma patients compared to healthy controls (22). Moreover, after subdividing the asthma groups into neutrophilic and eosinophilic asthma based on the percentage sputum neutrophils and eosinophils, increased miR-223 levels were found in neutrophilic asthma patients compared to healthy controls and eosinophilic asthmatics. Furthermore, miR-223 was negatively associated with FEV1, FEV1/FVC ratio, and positively associated with the percentage neutrophils in sputum (22). A recent study by Gomez et al. found almost similar results, miR-223 expression was increased in sputum of asthma patients and was associated with a neutrophilic asthma phenotype. Furthermore, miR-223 expression levels were also correlated with multiple features of severe asthma, bronchodilator response, and FeNO levels (29). In contrast, in a study using blood samples, miR-223 expression was lower in blood-derived T cells from mild to moderate asthma patients compared to healthy controls (21). Another study found that *in utero* exposure to cigarette smoke increases the risk of developing allergic sensitization, which was associated with lower levels of regulatory T cells and with high miR-223 expression in cord blood (34).

**Table 1:** Overview of miR-223 expression in asthma and COPD patients compared to controls.

**Feature**	**Participants**	**Controls**	**Findings**	**Method**	**References**
Asthma	8 mild atopic asthma patients	8 healthy controls	Not differentially expressed in bronchial biopsies	RT-qPCR	(26)
	12 mild to moderate asthma patients, 12 severe asthma patients	10 healthy controls	Not differentially expressed in bronchial biopsies	RT-qPCR	(27)
	16 steroid naive atopic asthma patients with bronchial hyperresponsiveness	12 healthy controls	↑ in bronchial airway epithelial cells	Micro-array	(28)
	16 eosinophilic asthma patients, 8 neutrophilic asthma patients	10 healthy controls	↑ in induced sputum supernatant of neutrophilic asthma patients	Micro-array	(22)
	29 eosinophilic asthma patients, 21 neutrophilic asthma patients	10 healthy controls	↑ in induced sputum supernatant of neutrophilic asthma patients	RT-qPCR	(22)
	62 asthma patients	9 controls	↑ in induced sputum of asthma patients	HiSeq Sequencing	(29)
	12 mild to moderate asthma patients	10 healthy controls	↓ in blood circulating naive and memory T cells	Micro-array	(21)
Smoke exposure	10 current smokers	10 non-smokers	↓ in bronchial airway epithelial cells	Micro-array	(30)
COPD	26 COPD patients	9 normal smokers	↑ in lung tissue	Micro-array/ RT-qPCR	(31)
	23 COPD patients	16 non-COPD controls	↑ in bronchoalveolar lavage fluid	Micro-array	(32)
	25 female COPD patients exposed to biomass smoke	25 female healthy controls exposed to biomass smokes	↑ in serum	Micro-array/ RT-qPCR	(33)

With regard to smoking and COPD, lower miR-223 expression levels have been observed in bronchial brushings from current smokers compared to never smokers (30). Ezzie et al. focusing on differentially expressed miRNAs in lung tissue of smokers with or without COPD, demonstrated higher miR-223 expression in COPD patients compared to smokers without COPD (31). Furthermore, higher levels of miR-223 were also measured in bronchoalveolar lavage fluid (BALF) obtained from COPD patients compared to non-COPD controls (32). In serum of women with COPD due to biomass smoke, miR-223 levels are higher compared to healthy controls exposed to biomass smoke (33).

Taken together, it is clear that miR-223 expression is differently expressed in obstructive lung disease and seems to be associated with neutrophilia (Table 1). However, thus far miR-223 expression was associated with an inflammatory phenotype in only two asthma studies (22, 29). In mild atopic asthma patients the increase in miR-223 could also originate from an eosinophilic component, since both neutrophils and eosinophils were present in the bronchial brushings (28). With regard to COPD, no data are available that link miR-223 expression to disease stage, inflammatory phenotype or presence of emphysema. Furthermore, (biomass) smoke can alter the expression of miR-223, which together with differences in examined samples and patient groups further adds to the complexity of the observed findings.

## Role of miR-223 in the Pathological Processes in Chronic Obstructive Lung Disease

**Figure 1:**
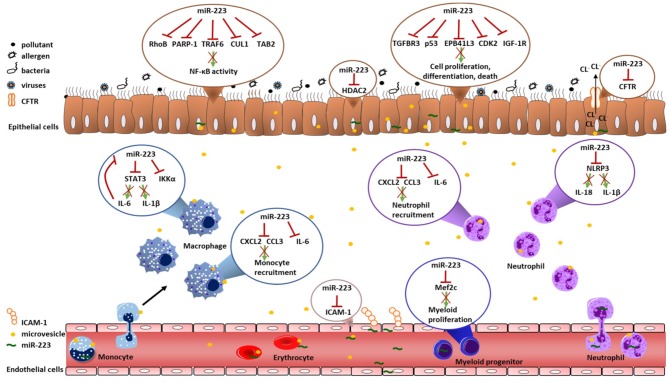
Overview of validated targets of miR-223. This overview illustrates all validated targets genes of miR-223 that could contribute to the pathogenesis of asthma and COPD. There were no studies that investigated validated targets of miR-223 in T-cells and eosinophils. The red cross indicates that miR-223 targets this gene and/or protein. CCL3, Chemokine (C-C motif) ligand 3; CUL1, cullin 1; CDK2, Cyclin-dependent kinase 2; CFTR, cystic fibrosis transmembrane conductance regulator; CXCL2, Chemokine (C-X-C motif) ligand 2; EPB41L3, erythrocyte membrane protein band 4.1 like 3; HDAC2, histone deacetylase 2; ICAM-1, Intercellular adhesion molecule 1; IGF-1R, insulin-like growth factor-1 receptor; IKKα, IκB kinase α; IL-6, Interleukin-6; Mef2c, Myocyte Enhancer Factor 2C; NLRP3, NOD-, LRR- and pyrin domain-containing protein 3; PARP-1, Poly [ADP-ribose] polymerase 1; RhoB, Rho-related GTP binding protein; STAT3, Signal transducer and activator of transcription 3; TAB2, TGF-Beta Activated Kinase 1 (MAP3K7) Binding Protein 2; TGFBR3, Transforming growth factor beta receptor III.

Both asthma and COPD are characterized by inflammatory responses and airway remodeling upon environmental exposures. In the paragraphs below we will describe how miR-223 can influence these processes. The role of miR-223 in regulating inflammation and cell proliferation, differentiation and death was reviewed before (15), but here we focus on the potential implications for asthma and COPD. We will use findings from *in vivo* murine models of infection and acute lung injury to speculate on the importance of miR-223 in asthma and COPD and highlight several of the miR-223 targets, which have been validated by luciferase assays (Table 2). Furthermore, we review findings obtained from *in vitro* studies, which are often within the context of cancer, but can also be relevant within the context of asthma and COPD. Table 3 gives a summary of the experiments done for miR-223 in each cell type and the expression of this target gene in asthma and/or COPD. Furthermore, Figure 1 shows an overview of the potential roles of miR-223 in the pathogenesis of asthma and COPD.

**Table 2:** Overview of the validated target genes of miR-223.

**Gene**	**Full gene name**	**Function**	**Species**	**References**
NFI-A	Nuclear Factor I A-type	Transcription factor	Human	(16)
LMO2	LIM-only protein 2	Erythropoeisis regulator	Human	(18, 19)
PARP-1	poly(ADP-ribose) polymerase 1	Regulator in cell death and NF-κB activity	Human	(35)
IKKα	IkB kinase α	Regulator of NF-κB pathway	Human	(36)
CUL1	Cullin 1	Protein degradation and ubiquitination	Human	(37)
TAB2	TGF-β-activated kinase 1/MAP3K7	Activation of MAP3K7 in the IL-1 signaling pathway	Human	(37)
NLRP3	NLR family pyrin domain containing 3	Pattern recognition receptor	Human	(24, 38, 39)
RhoB	Rho-related GTP-binding protein	Small signaling G protein	Human	(39)
CFTR	CF transmembrane conductance regulator	Chloride channel	Human	(40)
STAT3	Signal transducer and activator of transcription 3	Mediating anti- and pro-inflammatory responses	Human	(41)
CXCL2	C-X-C Motif Chemokine Ligand 2	Neutrophil chemo-attractant	Human	(42)
CCL3	C-C Motif Chemokine Ligand 3	Neutrophil chemo-attractant	Human	(42)
IL-6	Interleukin-6	Pro-inflammatory cytokine	Human	(42)
HDAC2	Histone deacetylase 2	Deacetylation of lysine	Human	(43)
Mef2c	Myocyte enhancer factor 2c	Promotes proliferation of myeloid progenitors	Human	(17)
IGF-1R	Insulin-like growth factor-1 receptor	Activates PI3K-Akt and mTOR signaling	Human	(17, 44–46)
TGFBR3	Transforming growth factor beta receptor III	TGF-β signaling	Human	(47)
CDK2	Cyclin-dependent kinase 2	Cell cycle	Human	(45)
p53	Tumor Protein P53	Tumorsuppressor gene	Human	(48)
EPB41L3	Erythrocyte Membrane Protein Band 4.1 Like 3	Cell proliferation, cell-cell contact	Human	(49)
ICAM-1	Intercellular adhesion molecule 1	Role in leukocyte trafficking	Human	(50)

*miR-223 validated targets by luciferase assays. The luciferase assay determine whether a specific miRNA controls the expression of a potential target gene. The 3′UTR of the potential target gene is cloned into the 3′ region of a luciferase reporter plasmid and co-transfected with the predicted miRNA, leading to repression of luciferase gene expression if the miRNA directly binds to the 3′-UTR*.

**Table 3:** Overview of miR-223 experiments and their expression in asthma and COPD.

**Overview of miR-223 experiments**				**Expression of target genes in asthma and COPD**		
**Expression of miR-223**	**Cell type**	**Condition**	**References**	**Asthma**	**COPD**	**References**
Downregulation miR-223	HBECs	↑ NF-kB activity	(37)	↑ NF-kb activity in HBECs	↑ NF-kb activity in HBECs	(51)
Overexpression of miR-223	HBECs	↓ PARP-1	(22)	↑ PARP-1 activity in PBMCs and lung tissue	↑ PARP-1 activity in blood	(52, 53)
Reduction of miR-223	Monocytes/Macrophages	↑ IKKα	(36)	No differences in PBMCs No differences in IKKα activity in PBMCs	No differences in PBMCs ↑ IKKα activity in PBMCs	(54)
Downregulation miR-223	Basal epithelial cells of zebrafish	↑ TRAF6	(37)	No differences in PBMCs	NA	(55)
Downregulation miR-223	HBECs	↑ CUL1	(37)	NA	NA	
Downregulation miR-223	HBECs	↑ TAB2	(37)	NA	NA	
Overexpression of miR-223	Murine neutrophils/A549 cells	↓ NLRP3	(24)	↑ NLRP3 in sputum of neutrophilic asthma patients	↑ NLRP3 in PBMCs No differences in HBECs	(56, 57)
Overexpression of miR-223	A549 cells	↓ RhoB	(39)	NA	NA	
Knock out of miR-223	Murine lung	↑ IL-6	(42)	↑ IL-6 BALF and sputum	↑ IL-6 BALF and sputum	(8)
Knock out of miR-223	Murine lung	↑ CCL3	(42)	↑ CCL3 in BALF and CD4+ T cells No differences in BALF ↑ CCL3 in CD8+ T-cells	↑ CCL3 in sputum	(58–62)
Knock out of miR-223	Murine lung	↑ CXCL2	(42)	NA	↑ CXCL2 in lung tissue	(63, 64)
Overexpression of miR-223	Murine macrophages	↓ STAT3	(41)	↑ STAT3 activity in airway smooth muscle cells	↑ STAT3 in lung tissue	(65–67)
Overexpression of miR-223	Human coronary artery endothelial cells	↓ ICAM-1	(50)	↑ ICAM-1 in blood, BALF and nasal lavage fluid	↑ ICAM-1 in lung tissue	(64, 68)
Overexpression of miR-223	human pulmonary artery endothelial cells (HPAEC)	↓ HDAC2	(43)	NA	↓ HDAC2 activity in lung tissue and alveolar macrophages	(69)
Knock out of miR-223	T cells	↓ Mef2c	(17)	↓ Mef2c in HBECs	NA	(70)
Overexpression of miR-223	Human adenocarcinoma	↓ IGF-1R	(44)	↑ IGF1 signaling in bronchial biopsies	↓ IGF-1 in serum ↑ IGF-1 in quadriceps	(44, 57, 61)
Knock out of miR-223	Neutrophils	↑ IGF-1R	(17)			
Overexpression of miR-223	Airway smooth muscle cells	↓ IGF-1R	(46)			
Overexpression of miR-223	Lewis lung carcinoma cells	↓ IGF-1R	(45)			
Overexpression of miR-223	A549 cells	↓ TGFBR3	(47)	↓ TGFBR3 in blood	↓ TGFBR3 in severe COPD patients	(71, 72)
Downregulation miR-223	SPC-A1 lung cancer cells	↑ TGFBR3	(47)			
Overexpression of miR-223	Human lung squamous cell carcinoma cells	↓ p53	(48)	NA	↑ p53 in lung tissue	(73, 74)
Overexpression of miR-223	Lewis lung carcinoma cells	↓ CDK2	(45)	NA	NA	

*BALF, Bronchoalveolar lavage fluid; CCL3, Chemokine (C-C motif) ligand 3; CDK2, Cyclin-dependent kinase 2; CUL, cullin 1; CXCL2, CXCL2, Chemokine (C-X-C motif) ligand 2; HBECs, human bronchial epithelial cells; HDAC2, histone deacetylase 2; ICAM-1, Intercellular adhesion molecule 1; IGF-1R, insulin-like growth factor-1 receptor; IKKα, IκB kinase α; IL-6, Interleukin-6; Mef2c, Myocyte Enhancer Factor 2C; NLRP3, NOD-, LRR- and pyrin domain-containing protein 3; PARP-1, Poly [ADP-ribose] polymerase 1; PBMCs, Peripheral blood mononuclear cell; RhoB, Rho-related GTP binding protein; STAT3, Signal transducer and activator of transcription 3; TAB2, TGF-Beta Activated Kinase 1 (MAP3K7) Binding Protein 2; TGFBR3, Transforming growth factor beta receptor III; TRAF6, TGF-beta activated kinase 1 binding protein 2*.

### Role of miR-223 in Inflammatory Responses

Several studies have demonstrated increased levels of miR-223 in blood samples and/or inflamed lung samples from mice that were exposed to allergens, bacteria, viruses, or fungi compared to samples from untreated mice, all relevant exposures for the development of obstructive lung disease (35, 42, 43, 75, 76). In this section we will overview the role of miR-223 in inflammatory responses.

#### Role of miR-223 in the Nuclear Factor Kappa B Pathway

The nuclear factor kappa B (NF-κB) pathway plays a major role in inflammatory responses by regulating cell survival, activation, and differentiation of immune cells. The activation of NF-κB induces expression of various pro-inflammatory genes and inflammasome activation (77). In bronchial biopsies obtained from asthma and COPD patients, NF-κB is activated upon exposure to various environmental triggers, leading to pro-inflammatory responses (51) and therefore the activation of NF-κB may be a critical component of the pathogenesis of both diseases.

Several studies suggest that miR-223 is a negative regulator of NF-κB signaling. While overexpression of miR-223 in human bronchial epithelial cells (HBECs) decreased NF-κB activity, downregulation of miR-223 in HBECs resulted in increased NF-κB activity after *Pseudomonas aeruginosa* stimulation (37). One of the targets of miR-223 involved in the activation of NF-κB pathway is poly (ADP-ribose) polymerase 1 (PARP-1). PARP-1 influences expression of various pro-inflammatory factors and chromatin remodeling, which promotes inflammation (78).

Overexpressing miR-223 in HBECs downregulates *PARP-1* expression (22). With regard to the pathogenesis of asthma, it was demonstrated that PARP-1 deficiency in OVA-induced allergic mouse models leads to a reduction of eosinophilia and Th2 cytokine production (79, 80). Furthermore, PARP-1 downregulates STAT-6, leading to downregulation of GATA-3, which is crucial in promoting type 2 differentiation and expression of IL-4, IL-5, and IL-13 (79). Of note, multiple *in vivo* and *in vitro* studies found contradicting results on PARP-1 inhibition and IL-17 levels, which is important for the recruitment of neutrophils (78). An *in vivo* study found elevated IL-17 levels in serum but decreased levels of GM-CSF, IL-22, and KC in BAL and increased levels of regulatory T cells in the spleen of PARP-1 deficient mice exposed to HDM. This suggest that PARP-1 depletion resulted in an anti-inflammatory effect (52). Similar to NF-κB, PARP-1 activity is increased in asthma patients, which was measured in peripheral blood mononuclear cells and lung tissue compared to controls (52). Based on the evidence that miR-223 is especially increased in sputum supernatant of neutrophilic asthma patients compared to healthy controls and eosinophilic asthma patients (22), we suggest that miR-223 acts as a modulator to limit PARP-1 expression, controlling eosinophilic, and neutrophilic responses in asthma. Therefore, it would be of interest to investigate the levels of PARP-1 in eosinophilic and neutrophilic inflammation in asthma and associate this with miR-223 expression. The activity of PARP-1 in COPD patients is systematically increased (53). *In vivo* experiments demonstrated that PARP-1 inhibition protects against emphysema and elastase induced-inflammation (81) and *in vitro* experiments showed that PARP-1 is activated upon cigarette smoke and oxidative stress (82). Furthermore, in an acute lung injury model with miR-223 deficiency, repression of PARP-1 reversed acute lung injury (35). However, the exact role of PARP-1 in COPD has not been well-addressed. Therefore, further studies are needed to identify the role and relationship of miR-223 and PARP-1 in COPD and its involvement in specific disease phenotypes.

Another target of miR-223 involved in the activation of NF-κB is IκB kinase α (IKKα), which together with IKKβ forms the IKK kinase complex (36). Amongst others, the exposure to bacteria and viruses can induce phosphorylation of the IKK kinase complex, resulting in NF-κB activation (83). Moreover, IKKα is a transcriptional regulator of Th17 differentiation by interacting with the RORγt transcription factor (84), and inhibition of IKKα results in lower levels of Th17 cells (85). During *in vitro* differentiation of human monocytes to macrophages, the reduction of miR-223 expression is associated with elevated IKKα levels (36). Upon TLR9 activation, higher levels of IKKα and reduced levels of several pro-inflammatory mediators have been found in bone marrow-derived miR-223 KO neutrophils compared to WT neutrophils (86). In asthma and COPD patients, IKKα levels were not different in peripheral blood mononuclear cells compared to healthy controls. However, higher p-IKKα levels were found in peripheral blood mononuclear cells of COPD patients and control smokers compared to asthma patients and non-smoking controls (54).

Recent studies showed that other miR-223 targets involved in the NF-κB activation are TGF-beta activated kinase 1 binding protein 2 (TRAF6), cullin 1(CUL1), and TGF-beta activated kinase 1 (MAP3K7) binding protein 2 (TAB2) (37, 87). Inhibition of miR-223 in basal epithelial cells from zebrafish induced TRAF6, CUL1, and TAB2 (37). TRAF6 is an intracellular signaling molecule mediating NF-κB activation by toll-like receptor activation of dendritic cells. Depletion of TRAF6 in dendritic cells resulted in higher infiltration of eosinophils in lung tissue and higher levels of IL-5, IL-13, and IGF-1 in an OVA-induced mouse model (88). This suggests that downregulation of TRAF6 by miR-223 contributes to Th2 responses. Moreover, in HBECs overexpression of miR-223 reduced NF-κB activity by targeting CUL1 and TAB2 (37). Furthermore, miR-223 knockdown in zebrafish leads to upregulation of *Cul1* expression, and subsequent higher neutrophil recruitment after wounding compared to controls (37). This indicates that in *in vivo* models miR-223 contributes to homeostasis of neutrophils and eosinophils.

In two different asthma cohorts, there were no significant differences in TRAF6 gene expression in PBMCs-derived from steroid naïve asthmatic children compared to healthy controls (55). For CUL1 and TAB2, it is unknown if there are any differences in expression between asthma, COPD patients, and healthy controls. Therefore, despite evidence from experimental studies, the effect of miR-223 on the target genes IKKα, TRAF6, and CUL1 in asthma and COPD patients, and whether their regulation contributes to the pathogenesis of these diseases, remains unclear.

#### Role of miR-223 in the IL-1 Signaling Pathway

NF-κB activation results in expression of several genes including the inflammasome NLR family pyrin domain containing 3 (NLRP3). Activation of the NLRP3 inflammasome leads to cleavage of pro-IL-1β and pro-IL-18 by caspase-1 into mature IL-1β and IL-18. IL-1β is a cytokine involved in the initiation and persistence of inflammation. NLRP3 can be activated by cigarette smoke, lipopolysaccharide (LPS), bacterial and viral exposures (89) and is identified in airway epithelial cells and peripheral blood neutrophils (90). Yuan et al. already highlighted that miR-223 represses NLRP3 function (15). Here we will focus on the role of miR-223 and NLRP3 in the context of asthma and COPD. In sputum of neutrophilic asthma patients excessive inflammasome activation and production of IL-1β have been found, which correlate with neutrophil counts in lung tissue and sputum, asthma severity, and steroid resistance in asthma (56, 90, 91). As well-increased gene expression of *NLRP3, IL-1*β, and *IL-18* has been found in PBMCs from COPD patients compared to smokers (92), while no differences of NLRP3, IL-1β, and IL-18 in bronchial epithelial were observed in severe stable COPD patients compared to current smokers (93), see also Table 3. This indicates that IL-1β and NLRP3 play an important role in neutrophilic asthma inflammation and in COPD.

Multiple *in vitro* and *in vivo* studies demonstrated that miR-223 targets NLRP3, resulting in reduced inflammation (24, 38, 39, 75). Overexpression of miR-223 in murine neutrophils reduces the activity of NLRP3, leading to less secretion of IL-1β (24), while miR-223 deficient mice have increased levels of NLRP3 in bone-marrow-derived neutrophils (75). However, NLRP3 or caspase-1 deficient mice still have acute pulmonary inflammation upon cigarette exposure (94). A recent study showed that overexpression of miR-223 in human adenocarcinoma alveolar basal epithelial cells reduced caspase-1, IL-1β, and IL-18 levels by targeting NLRP3 and rho-related GTP-binding protein (RhoB) after LPS stimulation (39). In addition, mice infected with *Klebsiella pneumoniae* and treated with microvesicles loaded with miR-223/miR-142 display reduced levels of caspase-1 activity in lung and reduced levels IL-1β and IL-18 in BALF, which the authors suggest is probably caused by targeting NLRP3 (38). Similar results were recently found in an acute and chronic liver injury mouse model, where a miR-223 mimic reduced NLRP3 and IL-1β expression in mice with endotoxin-induced acute hepatitis and diet-induced chronic fibrosis. Furthermore, this miR-223 mimic also led to less infiltration of macrophages and neutrophils in the liver, possibly due to the lower NLRP3 and IL-1β levels (95). An interesting finding is that *in vitro* stimulation of a bronchial epithelial cell line carrying a mutation in cystic fibrosis transmembrane conductance regulator (CFTR) gene with IL-1β induces miR-223 expression. Furthermore, they showed that CFTR is a validated target of miR-223 (40), which could be important since recently a link between CFTR and the chronic bronchitis phenotype in COPD was proposed (96).

In contrary to *in vitro* studies, the mechanisms mediating NLRP3 and IL-1β expression in obstructive lung diseases are more complex and the higher levels of miR-223 in neutrophilic asthma patients and COPD patients may not be sufficient to reduce pro-inflammatory gene expression as is observed in experimental studies.

#### Role of miR-223 During Infections

Infection can contribute to the development of asthma (97) and cause asthma and COPD exacerbations (97, 98). The impact of miR-223 deficiency on combatting infections, however differs depending on the model system and stimulus. *In vitro*, miR-223 deficient neutrophils have enhanced capacity to kill *Candidia albicans* compared to WT (wild type) neutrophils (17). However, *in vivo* infections with *mycobacterium tuberculosis* in miR-223 deficient mice leads to much higher mortality in contrast to WT littermates, while depletion of polymorphonuclear neutrophils upon infection prolonged the survival of miR-223 deficient littermates (42). In latter study, high bacterial burden, more neutrophils, and increased levels of the validated targets IL-6, C-C Motif Chemokine Ligand 3 (CCL3), and C-X-C Motif Chemokine Ligand 2 (CXCL2) were observed in the lung of miR-223 deficient mice compared to WT animals (42). Notably, in all models of infection and acute lung injury increased inflammation was found in miR-223 deficient mice compared to WT mice (17, 42, 75). This confirms the notion that miR-223 can have a protective role in lung inflammation upon microbial challenge, which could also be relevant in asthma and COPD.

As mentioned, miR-223 expression affected *IL-6, Cxcl2*, and *Ccl3* levels in the above described *mycobacterium tuberculosis* model (42). This is of interest, since IL-6 is also increased in BALF and induced sputum of asthmatics and COPD patients (8). IL-6 plays a role in the expansion of Th2 and Th17 cells, which play a role in airway inflammation in (severe) asthma and/or COPD. Furthermore, CCL3 (MIP-1α) and CXCL2 (MIP-2) are important mediators in asthma and COPD because they are chemoattractant for monocytes/macrophages and neutrophils. In asthma, the reports on CCL3 expression are not univocal, as increased as well as equal and reduced CCL3 levels have been found depending on the patient cohort, investigated sample or cell type (Table 3) (58–61). In COPD, higher levels of CCL3 were detected in sputum of COPD patients compared to healthy non-smokers and smokers, which correlate with IL-6 and sIL-6R levels in sputum of COPD patients (62, 99). This indicates that elevated miR-223 expression levels in asthma and COPD could thus influence monocytic inflammatory responses. With regard to CXCL2, little is known in asthma but it has been associated with a neutrophilic phenotype in mouse models (100). In COPD patients *CXCL2* gene expression is upregulated in lung tissue compared to controls (63, 64).

Another target of miR-223, which can also be activated by IL-6 and CXCL2 signaling, is STAT3. Overexpression of miR-223 in murine macrophages (Raw264.7) resulted in reduced levels of IL-6 and IL-1β upon LPS and poly(I:C) stimulation by targeting STAT3 (41). STAT3 signaling contributes to the induction and/or response of Th2 and Th17 cells (101–104). Asthma patients have increased STAT3 activity in airway smooth muscle cells (65) and COPD patients have increased STAT3 levels in lung tissue compared to controls (66, 67). Furthermore, a single nucleotide polymorphism (SNP) of STAT3 has been associated with atopic -but not with non-atopic asthma- in children (105). Whether miR-223 modulates STAT-3 in asthma and COPD and whether it is involved in neutrophilic or eosinophilic responses remains to be determined. Finally, miR-223 can also influence release of neutrophils into tissues by reducing the expression of cell adhesion molecule-1 (ICAM-1) in endothelial cells (50). ICAM-1 expression is increased in blood, BAL, and nasal lavage fluid of asthma patients and in lung tissue of COPD patients compared to controls (64, 68).

Taken together, miR-223 targets multiple genes that are involved in the recruitment and activation of neutrophils and monocytes/macrophages, and expansion of Th2 and Th17 cells.

#### The Influence of miR-223 on Epigenetic Alterations

Most studies demonstrated that miR-223 is involved in the NF-κB pathway and has an anti-inflammatory function. However, Leuenberger et al. showed that miR-223 can cause epigenetic changes by targeting histone deacetylase 2 (HDAC2), resulting in activation of the NF-κB pathway. HDAC2 is necessary to deacetylate histones resulting in suppression of several activated inflammatory genes. Increased expression of miR-223 and decreased expression of *HDAC2* were found in endothelial cells after stimulation with tumor necrosis factor-α and IL-1β. Furthermore, decreased levels of HDAC and HDAC2 activity were demonstrated in endothelial cells overexpressing miR-223, leading to unwinding of chromatin and activation of several proinflammatory cytokines and chemokines (43, 69). An *in vivo* study showed that heterozygous HDAC2 mutant mice have increased inflammatory responses, cellular senescence, airspace enlargement, and lung function decline after chronic cigarette exposure, compared to WT mice (106). Moreover, an earlier study found that COPD patients have lower HDAC2 activity in lung tissue and alveolar macropahges compared to healthy controls and that the severity of the disease is negatively correlated with HDAC2 activity (69). HDAC2 activity is an important regulator of corticosteroid sensitivity (107). COPD and asthma patients with lower HDAC2 activity compared to healthy controls are less sensitive to corticosteroids than patients with higher HDAC2 activity levels (108, 109). Overexpression of HDAC2 in alveolar macrophages can restore the corticosteroid sensitivity (69). This indicates that elevated miR-223 expression by e.g., neutrophils contributes to the insensitivity of corticosteroids in asthma and COPD patients by modulating HDAC2, which can worsen the severity of the disease. Notably, early-onset allergic eosinophilic asthmatics are generally corticosteroid-sensitive. It will be interesting to investigate how miR-223 expression correlates with HDAC2 expression in these patients.

#### Overall Conclusions for a Role of miR-223 in Inflammatory Pathways

Overall, previous paragraphs indicate that miR-223 mainly acts as an anti-inflammatory miRNA. Several studies showed that depletion of miR-223 resulted in enhanced inflammatory responses, while overexpression of miR-223 reduced pro-inflammatory responses. Moreover, miR-223 targets multiple genes that are involved in the NF-κB pathway, e.g., PARP1, IKKα, TRAF6, CUL1, and TAB2. Based on the evidence that miR-223 is increased in multiple samples from asthma and COPD patients, we propose a model where miR-223 acts as a protective miRNA to reduce/prevent pro-inflammatory responses. The increased levels of miR-223 could inhibit PARP-1 levels and modulate the balance of neutrophilic/eosinophilic responses. However, the higher levels of miR-223 in neutrophilic asthma patients may not be sufficient to reduce the levels of IL-6, CCL3, and CXCL2, which contribute to Th17 responses. Since most of the target genes of miR-223 are elevated in asthma and COPD, it might be that the protective effect of miR-223 is impaired or not sufficient to reduce the high inflammatory responses in asthma and COPD, possibly by changes in the local tissue compartments. Another explanation for increased expression of miR-223 target genes in asthma and COPD may be that miR-223 also targets HDAC2, which is important for corticosteroid sensitivity. In asthma and COPD patients with high miR-223 levels, the reduction in HDAC2 could lead to lower corticosteroid sensitivity and less reduction of pro-inflammatory cytokines and chemokines in response to corticosteroids. However, until now it is unknown if there is a direct link between miR-223 levels in the different disease phenotypes and corticosteroid sensitivity due to HDAC2.

Further studies should direct how miR-223 is involved in the pathogenesis of asthma and COPD, since the effects of altered miR-223 expression levels on pro-inflammatory responses remain enigmatic.

### Role of miR-223 in Cell Proliferation, Differentiation, and Death

In addition to the function of miR-223 in inflammatory processes, several studies have demonstrated that miR-223 contributes to cell proliferation, differentiation, and death. Cell dysfunction and death are also important features in asthma and COPD. In asthma dysregulated apoptosis has been found in T cells, eosinophils, and neutrophils (110). However, the exact contribution of apoptosis in airway epithelial cells to the pathogenesis of asthma is unclear. Oxidative stress induced by smoking can cause damage to the lung matrix and death of structural cells, leading to emphysema. In COPD patients increased apoptosis is present in the lungs, as well as decreased clearance of apoptotic cells (111).

#### Role of miR-223 in Cell Proliferation and Differentiation

The deficiency of miR-223 in a mouse model leads to hyperproliferation of neutrophils and enhanced differentiation of granulocyte-monocyte progenitor cells (112). MiR-223 targets myocyte enhancer factor 2c (*Mef2c*), a transcription factor that promotes proliferation of myeloid progenitors, indicating that miR-223 inhibits proliferation of myeloid progenitor cells. Depletion of *Mef2c* restores the hyperproliferation of neutrophils and expansion of granulocyte-monocyte progenitor cells (17). Interestingly, *Mef2c* is decreased in bronchial epithelial cells from mild and severe asthma patients compared to healthy controls (70), which could result from the elevated miR-223 levels in bronchial epithelial cells of asthma patients. Cell proliferation can also be controlled by insulin-like growth factor-1 receptor (IGF-1R). *In vivo* and *in vitro* studies demonstrated that IGF-1R is a validated target of miR-223 (17, 44–46). IGF-1 upon binding with IGF-1R results in activation of signaling pathway PI3K/Akt and mTOR that controls cell proliferation, growth, survival, and apoptosis. In eosinophil progenitors from miR-223 KO mice upregulated expression in IGF-1R has been found. This results in a delay in differentiation and growth of eosinophil progenitors (113), suggesting that changes in miR-223 can also modulate eosinophil function in asthma. Both in asthma and COPD abnormal IGF-1 signaling has been demonstrated (57). Asthma patients treated with ICS have reduced IGF-1 levels, which is associated with reduced airway wall thickness and inflammation (114). Furthermore, *in vivo* inhibition of *Igf1r* reduced airway inflammation, airway resistance, airway wall thickening, and eosinophil levels in blood and BALF in an OVA-induced mouse model (115) and reduced neutrophil and macrophage numbers in a bleomycin-induced mouse model (116). This indicates that miR-223 contributes to airway wall remodeling in asthma. COPD patients with acute exacerbation have decreased IGF-1 levels in serum compared to stable COPD patients and healthy controls (117), while in lung tissue mRNA levels of IGF-1 are increased in COPD patients compared to controls (63). It would be of interest to investigate if miR-223 levels are elevated upon ICS treatment in asthma and COPD.

#### Role of miR-223 in Cell Viability, Invasion, and Apoptosis

Besides cell proliferation and differentiation, several *in vitro* studies in lung cancer cells have investigated the role of miR-223 in cell viability and invasion and apoptosis. However, contradicting results have been found about the function of miR-223 in lung cancer cells, which is probably due to the use of different cell types. Two studies demonstrated that miR-223 promotes cell viability, invasion and reduces apoptosis in adenocarcinoma A549 cells (118) and in SPC-A1 lung cancer cells by targeting transforming growth factor β (TGF-β) receptor 3 (TGFBR3) (47). TGFBR3 is involved in TGF-β signaling, which regulates cell growth, differentiation, and development (47). Reduced expression of miR-223 was demonstrated in airway smooth muscle cells stimulated with TGF-β1 (46), while recently overexpression of miR-223 in DCs increased the levels of TGF-β upon LPS stimulation, which were associated with higher levels of IL-10 and more T regulatory cells (119). TGF-β and the receptors play a key role in airway remodeling in asthma and COPD. TGF-β signaling induces amongst others the expression of collagen and can cause the disruption of airway epithelial integrity, which contribute to epithelial-mesenchymal transition (120). In asthma patients, the expression of TGF-β1 is increased in bronchial biopsies and is associated with the severity of the disease (121). Increased TGF-β1 levels were also found in serum of COPD patients (122), however, in bronchial epithelial cells and alveolar macrophages of COPD patients TGF-β1 levels were decreased compared to control smokers (123).

Furthermore, in asthma and COPD patients several genetic variations (SNPs) were found for *TGFBR3* (124, 125). Decreased levels of TGFBR3 were measured in lungs from severe COPD patients with emphysema and in blood of asthma patients compared to controls (71, 72), which might be caused by increased levels of miR-223 in COPD and asthma patients.

Two other studies investigated the role of miR-223 in cell death. Overexpression of miR-223 in human lung squamous cell carcinoma cells inhibit migration and proliferation by targeting p53 (48). Furthermore, the growth rate of Lewis lung carcinoma cells is reduced upon miR-223 overexpression by targeting cyclin-dependent kinase 2 (CDK2). Similar to p53, CDK2 is involved in cell cycle arrest (45). Furthermore, a SNP of CDK2 has been associated with asthma (126). During homeostasis the expression of p53 is low. However, oxidative stress, which can be caused by smoking in airway epithelial cells (127), induces p53 expression and activity, resulting in cell apoptosis. Despite the elevated levels of miR-223 in COPD patients, the validated target p53 is still higher expressed in lung tissue of COPD patients compared to non-COPD controls, especially in smoking COPD patients (73, 74). This suggests that miR-223 could be less sufficient in reducing apoptosis by targeting p53 in COPD patients compared to non-COPD controls.

Overall, miR-223 can target several genes that are involved in cell proliferation, differentiation, viability, invasion, and death by targeting *Mef2c, IGF-1R, TGFBR3, CDK2*, and *p53*, genes that have been implicated in the pathogenesis of asthma and/or COPD. However, the function of miR-223 is cell type and tissue dependent. Since structural cells barely express miR-223, the functional differences of miR-223 might be caused by the transfer of miR-223 from myeloid cell to structural cells.

## Transfer of miR-223 to Recipient Cells

During the last decade, it has become clear that miRNAs can be transferred to recipient cells by extracellular vesicles and high-density lipoproteins, influencing biologic processes in recipient cells (128). Extracellular vesicles contain proteins, lipids, and DNA and RNA molecules, including miRNAs, which can be important non-invasive biomarkers for asthma and COPD (129). Recent studies have shown that inflammatory cells can transfer miR-223 to lung epithelial cells via extracellular vesicles. During homeostasis, the expression of miR-223 is low in alveolar and bronchial epithelial cells, however during lung inflammation miRNAs can transfer in extracellular vesicles derived from polymorphonuclear neutrophils to alveolar epithelial cells, increasing miR-223 levels in epithelial cells (35). A functional study on miR-223 transfer in extracellular vesicles found that miR-223 is still functional active after uptake in recipient cells (130). Furthermore, dramatically increased extracellular vesicle numbers were measured in BALF and serum of mice treated with LPS or *Klebsiella pneumoniae*. These extracellular vesicles are derived from lung macrophages (38). Liang et al. found that activated platelets also release large amounts of extracellular vesicles that contain high levels of miR-223 and can transfer to human adenocarcinoma A549 cells (49). High-density lipoproteins can also transfer miR-223 into endothelial cells (50). A recent study showed that severe asthma patients have less mature miRNA in extracellular vesicles-derived from BALF and that the miRNA profiles are altered compared to healthy controls, with 90 downregulated miRNA (including miR-223) in asthma. Furthermore, increased expression levels of predicted target genes of these 90 miRNAs were demonstrated, including genes involved in inflammation and remodeling, such as the TGF-β receptors (131). Extracellular vesicles derived from mild asthma patients appear to have a different miRNA profile than those from severe asthma patients, since comparison of miR-223 expression between extracellular vesicles derived from mild asthma patients compared to healthy controls showed no differences (132). Furthermore, BALF from allergen-exposed mice contains higher miR-223 levels compared to BALF from control mice (133). Moreover, cigarette smoke exposure can change the composition of extracellular vesicles derived from HBECs (134). Moreover, COPD patients with acute exacerbation release more extracellular vesicles compared to non-smoking healthy controls (135). This indicates that upon stress extracellular vesicles are altered and might contribute to pro-inflammatory responses. Although miR-223 can be transferred in extracellular vesicles or high-density lipoproteins to recipient cells to control or alter biological functions, further study is required to identify the therapeutic potential of extracellular vesicles in asthma and COPD.

## Future Experiments

Despite the fact that miR-223 is one of the best studied miRNAs in literature, its functional role in pathogenesis of asthma and COPD remains incompletely understood. One important topic that needs to be addressed further is the identification of precise targets of miR-223 in health and disease and to discriminate in which cellular compartments these targets are altered in disease. In this review we only focused on validated targets, implicating that probably many other miR-223-controlled genes that are relevant for asthma and/or COPD were not considered. A second important area that requires future research is the discrimination between different diseases phenotypes; e.g., eosinophilic, neutrophilic, paucigranulocytic asthma and the involvement of different target such as PARP-1 and NLRP3 in the determination of these phenotypes.

In addition to investigating the expression of miR-223 and its targets in samples from well-characterized patient groups, also the use of *in vivo* murine models can provide more insight into the role of miR-223 in asthma and COPD. MiR-223 deficient mice can be tested in different asthma and COPD mouse models to better understand how miR-223 contributes to asthma and COPD. Here, the classical models of sole allergen expose or combined exposures with air pollution or microbial triggers such as LPS could be applied, which could mimic a predominant eosinophilic or a mixed neutrophilic/eosinophilic phenotype in asthma (136, 137). For COPD, mouse models with exposure to cigarette smoke, LPS or elastase can be used (138, 139). These studies can help to investigate the role of miR-223 in different asthma and COPD phenotypes when using knockouts for miR-223. However, an important point of consideration when using miR-223-deficient mice is the involvement of miR-223 in hematopoietic cell development, which can complicate evaluation of inflammatory processes. A complementary approach is therefore the use of miR-223 mimics or antagomirs to up- or downregulate miR-223 expression in a specific tissues, such as the lung via inhalation. The use of mimics and antagomirs can unravel the role of miR-223 in the pathogenesis of asthma and COPD, but also in in other diseases (140).

## Diagnostic and Therapeutic Opportunities of miR-223 in Obstructive Respiratory Diseases

Based on the findings of the studies described above, we suggest that it requires further investigation to determine if miR-223 can be a new diagnostic and/or therapeutic opportunity. The high and stable secretion of miR-223 in bodily fluids makes it a potential biomarker in asthma and COPD. The findings for miR-223 in sputum of neutrophilic asthma patients and the correlation between miR-223 expression and lung function (22) and bronchodilator response (29) suggest that miR-223 could be a diagnostic tool to determine asthma severity. However, miR-223 cannot be linked to a particular phenotype as higher levels of miR-223 were found in bronchial airway epithelial cells from atopic asthma patients with bronchial hyperresponsiveness as well (28). Ideally such a biomarker should predict the disease severity or phenotype in e.g., blood plasma or serum, since sputum inductions are not part of standard clinical practice. It therefore requires further investigation whether the correlations between miR-223 expression, inflammatory phenotype and disease severity are similar in other tissue compartments.

Overall, most of the *in vitro* studies have indicated an anti-inflammatory role of miR-223. Therefore, despite of the increased levels of miR-223 in asthma and COPD patients, we suggest that overexpressing miR-223 (e.g., by a miRNA mimic), rather than inhibiting miR-223, could be a therapeutic approach in asthma and COPD. Recent studies in cancer showed that there are multiple options to deliver the miRNA of interest, e.g., DNA plasmids, lipid vesicles, nanoparticles, or viral vectors (141). In the context of asthma and COPD, overexpression miR-223 in bronchial epithelial cells can be an option. However, main challenges of miRNA therapy are accuracy and efficiency of delivery. Since the miRNAs need to reach the right target cells, their uptake must be efficient and the correct genes must be targeted, without eliciting unwanted innate immune responses (142). Therefore, many hurdles have to be taken to consider miR-223 as a therapeutic option for asthma and COPD.

## Conclusions

In this review, we summarized current knowledge on miR-223 and how this miRNA could be involved in the development and pathogenesis of chronic obstructive airway diseases. In asthma patients, higher miR-223 expression levels in bronchial airway epithelial cells and in induced sputum have been observed, while COPD patients have higher miR-223 expression levels in lung tissue compared to healthy controls. *In vivo* and *in vitro* experiments showed that miR-223 is involved in pathways associated with cell proliferation, differentiation, and death and remodeling, however, the exact role is still unclear. Furthermore, miR-223 affects neutrophil function. Higher levels of miR-223 protect mice from lung inflammation, probably by reducing the activity of the NF-κB pathway. Therefore, most data point to an anti-inflammatory role of miR-223, and thus suggest that increased miR-223 levels in the airways try to counteract the ongoing inflammation. It might be that *in vivo* miR-223 is not sufficient to reduce the strong inflammatory responses in asthma and COPD or that epithelial cells from asthma and COPD patients are less able to take up miR-223. In conclusion, miR-223 is a fascinating miRNA and additional studies are required to unravel its exact role in the pathogenesis of asthma and COPD.

## Author Contributions

MR and TM contributed conception and design of the review. MR drafted and finalized the manuscript. KB, IH, and TM revised the manuscript and provided critical advice on the content of the manuscript. All authors contributed to manuscript revision, read, and approved the submitted version.

## Conflict of Interest

TM has participated in an advisory board from GlaxoSmithKline. TM is shareholder of Oryzon Genomics and of Mendelion Lifesciences SL. The remaining authors declare that the research was conducted in the absence of any commercial or financial relationships that could be construed as a potential conflict of interest.

## Abbreviations

BALF: Bronchoalveolar lavage fluid
C/EBPα: CCAAT enhancer protein α
CCL3: C-C Motif Chemokine Ligand 3
CDK2: Cyclin-dependent kinase 2
CFTR: Cystic fibrosis transmembrane conductance regulator
COPD: Chronic obstructive pulmonary disease
CUL1: Cullin 1
CXCL2: C-X-C Motif Chemokine Ligand 2
EPB41L3: Erythrocyte membrane protein band 4.1 like 3
HBECs: Human bronchial epithelial cells
HDAC2: Histone deacetylase 2
ICAM-1: Intercellular adhesion molecule 1
ICS: Inhaled corticosteroids
IGF-1R: Insulin-like growth factor-1 receptor
IKKα: IκB kinase α
IL-6: Interleukin-6
LPS: Lipopolysaccharide
Mef2c: Myocyte enhancer factor 2c
MiRNAs: MicroRNAs
mRNA: Messenger RNA
NF-κB: Nuclear factor kappa B
NFI-A: Nuclear factor I A-type
NLRP3: NLR family pyrin domain containing 3
OVA: Ovalbumin
PARP-1: Poly (ADP-ribose) polymerase 1
PBMCs: Peripheral blood mononuclear cell
RhoB: Rho-related GTP binding protein
RT-qPCR: Quantitative real time polymerase chain reaction
SNP: Single nucleotide polymorphism
STAT3: Signal transducer and activator of transcription 3
TAB2: TGF-beta activated kinase 1 (MAP3K7) binding protein 2
TGF-β: Transforming growth factor β
TGFBR3: TGF-β receptor 3
Th2: T helper 2
TRAF6: Tumor necrosis factor receptor associated factor 6.
